# Patterns of ocular inflammation in patients with miliary tuberculosis

**DOI:** 10.12688/f1000research.11035.1

**Published:** 2017-04-03

**Authors:** Salil Mehta

**Affiliations:** 1 Department of Ophthalmology, Lilavati Hospital and Research Center, Bandra Reclamation, Mumbai, 400050, India

**Keywords:** ocular tuberculosis, miliary tuberculosis, tubercles, ocular inflammation

## Abstract

Background: Ocular morbidity associated with systemic tuberculosis is common. The clinical picture varies from anterior uveitis, intermediate uveitis and posterior uveitis to even panuveitis. There is little data on the correlation between specific systemic presentations and the ocular inflammation. We conducted a retrospective review of the ocular findings in the case records of patients admitted with a diagnosis of miliary tuberculosis. These patients were then referred for a more detailed ophthalmic evaluation.

Methods: We analysed the case records of patients with a clinical diagnosis of miliary tuberculosis over a 10-year period at Lilavati Hospital  and Research Center, Mumbai.

Results: In total, 11 immunocompetent patients were identified. All 22 eyes showed normal findings on slit lamp examination. Dilated fundus examination showed single or multiple tubercles. In our cohort, the ocular findings were exclusively in the form of choroidal tuberculosis, either unilaterally or bilaterally. Slit lamp examination revealed no anterior segment inflammation

Conclusions: We suggest that this pattern of choroidal/retinal tuberculosis in the absence of anterior and intermediate segment inflammation is specific for miliary tuberculosis and may be related to a specific immune response.

## Introduction

Tuberculosis is a significant cause of uveitis, with published literature describing a spectrum of ocular inflammation that includes anterior uveitis, intermediate uveitis and posterior uveitis or even panuveitis in patients with different presentations of systemic tuberculosis. However, little data exists on the correlation between specific systemic presentations and any ocular inflammation that may co-exist.

Miliary tuberculosis is a specific systemic presentation that is commonly associated with ocular inflammation. We conducted a retrospective observational study of patients admitted with a diagnosis of miliary tuberculosis, to assess the specific patterns of any associated ocular inflammation.

## Methods

The study was conducted at Lilavati Hospital (Mumbai, India), which is a private tertiary healthcare facility. The Institutional Ethics Committee of Lilavati Kirtilal Mehta Medical Trust Research Centre approved this study for publication (09/02/2017).

We defined miliary tuberculosis as “tiny, discrete, widespread and uniform-sized lung opacities 2 mm or less in diameter (millet grains) on X ray or CT scan”. We retrieved the records of matching patients from 2006–2016 and excluded records of patients with HIV infection, autoimmune disease or on immunosuppressive therapy.

As part of a regular protocol that recognizes the diagnostic value of fundoscopy, all patients with a probable diagnosis of miliary tuberculosis were referred for an ophthalmic evaluation. All patients or their next of kin provided written informed consent for an ocular evaluation. Following this consent, patients underwent assessment of best-corrected visual acuity, slit lamp examination, and dilated indirect fundus examination and intraocular pressure assessment with an applanation tonometer. Patients unable to undergo a full evaluation underwent dilated indirect fundus examination at the bedside but were scheduled to complete the evaluation once their systemic status had improved. All patients who gave their written informed consent underwent fundus photography or optical coherence tomography (OCT) studies for documentation. All patients underwent both tests.

The following additional data was retrieved from patient records: age, sex, findings of chest X rays, CT scans (chest, brain or abdominal) and laboratory data (complete and differential blood counts, mantoux testing, renal and liver function tests at the least). Microbiological data included blood cultures (aerobic, anaerobic, mycobacterial cultures) and cultures of sputum studies.

## Results

In total, 11 immunocompetent patients were identified. These included 5 males and 6 females with ages ranging from 4 to 73 years (mean 42.5). All were ethnically Indian and their socio-economic status varied from the indigent residing in high-density tenements/slums to the affluent. Sources of referral included transfer from neighborhood facilities or from family physicians.

The common modes of clinical presentation of miliary tuberculosis included persistent fever, (7 patients: 4 males and 3 females with ages ranging from 4–73 years) or sepsis (4 patients: 3 females and 1 male with ages ranging from 16–71 years). 6 patients underwent a detailed evaluation soon after admission. The remaining 5, who were significantly ill, underwent only dilated fundus examination at that time.

The eyes of all 11 patients were analyzed (22 eyes in total). All patients were visually asymptomatic. Visual acuity studies were available for 6 of the 11 patients and were normal with 6/6 best-corrected visual acuity. All 22 eyes gave normal findings (no cells/flare) on slit lamp examination. Dilated fundus examination showed single or multiple tubercles bilaterally in 7 patients and unilaterally in 4 patients. No vitritis or raised intraocular pressure was seen in any patient. ( Table 1).

**Table 1.** The clinical and ocular findings of patients with miliary tuberculosis in our cohort. ARDS: Acute Respiratory Distress Syndrome; CNS: Central Nervous System; RE: right eye; LE: left eye; BE: both eyes.

Sr.No	Age/Sex	Diagnosis	Vision (RE)	Vision (LE)	Anterior Segment	Posterior Segment
1	44/f	Miliary tuberculosis (ARDS)	NA	NA	Normal (BE)	Tubercles (BE)
2	42/f	Miliary tuberculosis (ARDS)	6/6	6/6	Normal (BE)	Tubercles (BE)
3	73/m	Miliary tuberculosis	NA	NA	Normal (BE)	Normal (RE) Tubercles (LE)
4	54/m	Miliary tuberculosis	NA	NA	Normal (BE)	Normal (RE) Tubercles (LE)
5	57/m	Miliary and CNS tuberculosis	6/6	6/6	Normal (BE)	Tubercles (BE)
6	4/f	Miliary and CNS tuberculosis	NA	NA	Normal (BE)	Tubercles (BE)
7	71/m	Miliary and CNS tuberculosis	6/6	6/6	Normal (BE)	Tubercles (BE)
8	16/f	Miliary tuberculosis	6/6	6/6	Normal (BE)	Tubercles (BE)
9	14/f	Miliary tuberculosis	6/6	6/6	Normal (BE)	Tubercles (BE)
10	16/f	Miliary tuberculosis	NA	NA	Normal (BE)	Normal (RE) Tubercles (LE)
11	71/m	Miliary and CNS tuberculosis	6/6	6/6	Normal (BE)	Normal (RE) Tubercles (LE)

4 patients (3 females and 1 male, ages ranging from 16–71 years) gave consent for both fundus photography and OCT to be performed, and both tests were carried out.

Additionally, patients (2 females, 42 and 44 years old) had additional signs of acute respiratory distress syndrome (ARDS). 4 patients (3 men and 1 women; ages ranging from 4–71) had central nervous system (CNS) granulomas found in the frontal, parietal or temporal regions.

A standard therapy of INH, rifampicin, ethambutol and pyrazinamide was given. Systemic steroids were used at the discretion of the treating physician. Follow-up was available for 3–12 months for 4 patients (3 female and 1 male, ages ranging from 16 to 71, mean 30.5) until the choroidal tubercles were healed.

The clinical and ocular data of these patients is available in Dataset 1 ^1^.

Clinical and ocular data of study patients Click here for additional data file. Copyright: © 2017 Mehta S 2017 Data associated with the article are available under the terms of the Creative Commons Zero "No rights reserved" data waiver (CC0 1.0 Public domain dedication).

## Discussion

Of the 1.7 billion individuals infected with tuberculosis, only 10 % will develop an active infection in their lifetime, due to a protective immune response that can also be damaging to the tissues and is responsible for the clinical picture seen during active disease. The various clinical presentations are the result of a complex interaction between immune cells, secreted cytokines and varying combinations of systemic Th1 and/or Th2 responses.

Miliary tuberculosis accounts for 2% of all new cases of tuberculosis and approximately 20% of all extrapulmonary tuberculosis cases ^2^. It is a potentially fatal form of disseminated TB that follows from massive hematogenous dissemination. Its etiopathogenesis involves immune responses skewed towards a Th2 response that inhibits protective responses (granuloma formation), and this may permit widespread dissemination ^3^.

The ocular correlates of the systemic picture have been less well studied. Mehta analyzed the PET/CT scans of 27 patients in total; 13 with anterior uveitis, 7 with intermediate uveitis, 6 with pan-uveitis, 2 with vasculitis and 1 with multifocal serpiginous-like choroidopathy. 14 showed metabolically active, largely mediastinal, lymphadenopathy, and lung parenchymal disease was only rarely seen. The author postulated that a specific immune response to mycobacteria in the target tissues was responsible for this pattern of disease; i.e. systemic lymph node tuberculosis with its ocular correlate in the form of uveitis, with marked anterior and intermediate inflammation ^4^.

In our cohorts, who differ significantly in their systemic presentation from the previously mentioned study, the ocular findings were exclusively in the form of choroidal tuberculosis, either unilaterally or bilaterally. Slit lamp examination revealed a marked absence of anterior or intermediate segment inflammation. All the patients had evidence of tubercles, thus confirming the diagnostic role of fundoscopy, but a larger cohort is needed to confirm the absence of anterior segment inflammation.

We suggest that this pattern of choroidal/retinal tuberculosis in the absence of anterior and intermediate segment inflammation is specific for miliary tuberculosis and may be due to a specific immune response. A larger study that assesses the CD4 and CD8 counts and the cytokine profile is needed to elucidate the exact nature of the immune response responsible.

## Data availability

The data referenced by this article are under copyright with the following copyright statement: Copyright: © 2017 Mehta S

Data associated with the article are available under the terms of the Creative Commons Zero "No rights reserved" data waiver (CC0 1.0 Public domain dedication). 

Dataset 1: Clinical and ocular data of study patients.

DOI, 10.5256/f1000research.11035.d155310 ^1^
