# Supplementary material for: Patterns of ocular inflammation in patients with miliary tuberculosis
Source: F1000Res. 2017 Apr 3;6:412. [Version 1] doi: 10.12688/f1000research.11035.1 (PMC5405790; doi:10.12688/f1000research.11035.1)
Supplement: Peer review history

# Peer review: Referee response for version 1

**DOI:** 10.5256/f1000research.11903.r21514

**Published:** 2017-05-11

**Authors:** Padmamalini Mahendradas

**Affiliations:** 1 Department of Uveitis and Ocular Immunology, Narayana Nethralaya, Bengaluru, Karnataka, India

1. The author has reported that the choroidal tubercle is a characteristic finding in cases of military tuberculosis in an immunocompetent cases. Unlike other types of systemic tuberculosis (for example, pulmonary, lymphadenitis) which could be associated with several different ocular manifestations, it is interesting that choroidal tubercle is the only presentation observed in military tuberculosis. 
2. Providing the fundus photographs and optical coherence tomography images of choroidal tubercles along with radiological images of military tuberculosis cases would add additional value to the manuscript.
3. The author has mentioned that systemic steroids were used at the discretion of the treating physician. Addition of treatment details in the Data set 1 will give a more clear picture regarding the  management  of individual cases  to the readers.

 

I have read this submission. I believe that I have an appropriate level of expertise to confirm that it is of an acceptable scientific standard.

# Peer review: Referee response for version 1

**DOI:** 10.5256/f1000research.11903.r22605

**Published:** 2017-05-10

**Authors:** Somasheila I. Murthy

**Affiliations:** 1 LV Prasad Eye Institute, Hyderabad, Telangana, India

The study provides information on the patterns of ocular inflammation in patients with milliary tuberculosis over a 10-year period. The finding of choroidal tubercles in the presence of normal visual acuity and in the absence of associated anterior chamber inflammation or significant vitritis highlights the importance of screening posterior segment in patients with milliary tuberculosis. The examination can be easily done as a bed side procedure.

 A few points mentioned below, if further analyzed, can help to improve the understanding of disease behavior in these patients.

  

1. All patients had choroidal lesions detected on fundus examination. It would have been better to know at what time points the patients were examined. Were any of these patients already on anti-tubercular therapy (ATT) at the time of presentation or were started on it only after the presentation.
2. The course and time of resolution of choroidal tubercles following initiation of ATT using OCT imaging at least in patients who were ambulatory will be an important addition.
3. Did any of the patients have persistent lesions after the completion of ATT requiring prolonged continuation of ATT or some additional form of treatment?
4. Did any of the patient have a resurgence of the disease following discontinuation of ATT, after an initial cure. Information regarding the recurrence pattern can further provide an insight into the etiopathogenesis of this manifestation.

I have read this submission. I believe that I have an appropriate level of expertise to confirm that it is of an acceptable scientific standard.

# Peer review: Referee response for version 1

**DOI:** 10.5256/f1000research.11903.r21512

**Published:** 2017-04-21

**Authors:** Reema Bansal

**Affiliations:** 1 Department of Ophthalmology, Advanced Eye Center, Postgraduate Institute of Medical Education and Research, Chandigarh, Haryana, Punjab, India

1. The authors report the ocular profile of a rare but an important systemic disease, which adds relevant information to the literature. The study highlights the choroidal tubercles as hallmarks of miliary TB.
2. Providing fundus photographs would add value to the manuscript.
3. Four patients did undergo both fundus photography and OCT. But the authors haven’t described the OCT findings in any of these.
4. Another reference may be added; Sharma *et al. *2012. 

I have read this submission. I believe that I have an appropriate level of expertise to confirm that it is of an acceptable scientific standard.

## References

[1] SharmaSKMohanASharmaA: Challenges in the diagnosis & treatment of miliary tuberculosis. *Indian J Med Res*.2012;135(5) :703-30 22771605PMC3401706

# Peer review: Referee response for version 1

**DOI:** 10.5256/f1000research.11903.r21905

**Published:** 2017-04-18

**Authors:** Jyotirmay Biswas

**Affiliations:** 1 Vision Research Foundation, Sankara Nethralaya, Chennai, Tamil Nadu, India

Author reported the pattern of ocular lesions in patients with miliary tuberculosis. He analysed the record of the patients with the clinical diagnosis of miliary tuberculosis over ten years period seen in a hospital of Western India.

 All the patients were immunocompetent, had normal anterior segment findings but fundus examination showed single or multiple tubercles in the choroid. This is a rare cohort of patients of miliary tuberculosis who have got an ophthalmic examination done.

 It would have been better if authors could put some photograph of the fundus of these patients showing choroidal tubercles. We have studied 1005 patient of systemic tuberculosis in TB hospital in 1985 and found in 1.39% patients had ocular tuberculosis and only one patient had miliary tubercles in choroid (Biswas *et al.* 1996). New imaging system like optical coherence tomography particularly enhanced-depth and swept source of optical coherence tomography could show, the lesion location elegantly.

 Interestingly these lesions are in the periphery, not in the macular area and the patients preserve good visual function. I feel this article will be good addition to ophthalmic tuberculosis and systemic tuberculosis literature.

I have read this submission. I believe that I have an appropriate level of expertise to confirm that it is of an acceptable scientific standard.

## References

[1] BiswasJBadrinathSS: Ocular morbidity in patients with active systemic tuberculosis. *Int Ophthalmol*.19(5) :293-8 886481310.1007/BF00130924
